# Spatio-temporal patterns and characteristics of swine shipments in the U.S. based on Interstate Certificates of Veterinary Inspection

**DOI:** 10.1038/s41598-019-40556-z

**Published:** 2019-03-08

**Authors:** Erin E. Gorsich, Ryan S. Miller, Holly M. Mask, Clayton Hallman, Katie Portacci, Colleen T. Webb

**Affiliations:** 1 Department of Biology, Colorado State University, Fort Collins, CO USA; 2 Graduate Degree Program in Ecology, Colorado State University, Fort Collins, CO USA; 3 The Zeeman Institute: Systems Biology and Infectious Disease Epidemiology Research (SBIDER), University of Warwick, Coventry, UK; 4 School of Life Sciences, University of Warwick, Coventry, UK; 5 USDA APHIS Veterinary Services, Center for Epidemiology and Animal Health, Fort Collins, CO USA

## Abstract

Domestic swine production in the United States is a critical economic and food security industry, yet there is currently no large-scale quantitative assessment of swine shipments available to support risk assessments. In this study, we provide a national-level characterization of the swine industry by quantifying the demographic (i.e. age, sex) patterns, spatio-temporal patterns, and the production diversity within swine shipments. We characterize annual networks of swine shipments using a 30% stratified sample of Interstate Certificates of Veterinary Inspection (ICVI), which are required for the interstate movement of agricultural animals. We used ICVIs in 2010 and 2011 from eight states that represent 36% of swine operations and 63% of the U.S. swine industry. Our analyses reflect an integrated and spatially structured industry with high levels of spatial heterogeneity. Most shipments carried young swine for feeding or breeding purposes and carried a median of 330 head (range: 1–6,500). Geographically, most shipments went to and were shipped from Iowa, Minnesota, and Nebraska. This work, therefore, suggests that although the swine industry is variable in terms of its size and type of swine, counties in states historically known for breeding and feeding operations are consistently more central to the shipment network.

## Introduction

Swine production in the United States is a 19-billion-dollar industry encompassing over 63,000 operations and 71 million head^1^. The industry is becoming both increasingly specialized and connected to the global market^2^. Traditional small-scale farrow-to-finish operations are being replaced by large, single-phase operations with high densities of animals and lower production costs^3^. This growing industry has seen improved efficiency and profits in association with increased specialization. Yet, the resulting changes in the number, size, and national-level patterns of swine shipments requires characterization because the patterns of live animal shipments within the industry influence infection risk, economic development, and animal welfare^4,5^. For example, the movement of swine between premises has been shown to influence the introduction and spread of disease (e.g. classical swine fever^6^, foot-and-mouth disease^7^, porcine epidemic diarrhoea^8,9^, porcine respiratory and reproductive syndrome^10^). Risk assessments to support this growing industry, therefore, require accurate information on how shipments connect the swine industry and how shipment patterns change over time.

Network analyses facilitate the characterization of livestock shipment patterns and broaden our understanding of shipments beyond pairwise interactions to the network as a whole^11,12^. Network analyses have been used to characterize national-scale swine shipments in other countries^13–17^ and national-scale cattle shipments in the United States^18,19^. Recent work has also demonstrated the utility of network analyses to improve allocation of surveillance resources^20,21^, improve disease trace investigations^22^, and provide information on how livestock disease might spread through shipment networks^23,24^. As a result, livestock shipment networks are increasingly being used to better understand the underlying drivers of movement and evaluate how this information may be used to improve outbreak response and mitigate pathogen transmission.

The most comprehensive data available to characterize swine shipments at the national scale in the United States are Interstate Certificates of Veterinary Inspection (ICVI). ICVIs regulate the interstate movement of animals across the United States and certify that an accredited veterinarian has inspected the health of the animal and confirmed the disease testing requirements of the destination state. ICVIs are required for non-slaughter shipments that cross state lines, including shipments to sales, markets or shows. They detail the origin and destination address of the shipment and often provide information on the shipment’s size and purpose^25^. As a result, they are the most complete and consistently collected data on livestock shipments in the United States and serve as one of the principle data sources when conducting disease trace investigations to control outbreaks.

ICVI-based analyses and their characterization of domestic swine shipments represent an improvement over previous characterizations of the swine industry because they allow national-scale analyses using data on premises-to-premises shipments. Previous characterizations of U.S. swine shipments have either quantified premises-level shipments at a smaller scale^26,27^ or used state-level summaries of shipments at the national-scale^28^. One study used premises-level data to quantify shipment information for a single multi-site production system in a single state^10^. This study provided detailed within-state shipment information, but due to confidentially concerns, the number and locations of the production sites were omitted, precluding the use of similar datasets for national-scale risk analysis. An additional study by the United States Department of Agriculture (USDA) Economic Research Service quantified state-level estimates of domestic swine exports based on ICVI data from 2001^28^. Although this summary information facilitates national-scale analysis^9^, it does not provide potentially important information on heterogeneities in shipments essential for understanding of livestock movement and disease.

In this study, we compile and analyze swine ICVI data from 2010 and 2011 for three primary objectives. First, we characterize the age, sex, and production diversity within ICVI shipments that may be important for disease surveillance, trace investigations, and an improved understanding of industry production practices. Second, we use network analyses similar to those implemented for U.S. cattle shipment networks^18,19^ to characterize the national-scale patterns of county-to-county domestic swine shipments, how they vary by region, and their stability over time. We also quantify network metrics that inform county-level infection risk and network-level infection dynamics, both of which may be important for allocation of disease surveillance and predicting disease spread. Third we evaluate ICVIs as a dataset by comparing ICVI networks metrics to several additional datasets.

## Methods

### Data collection and entry

ICVI records are maintained and stored largely as paper records by the state departments of agriculture or the state veterinarian’s office. We compiled ICVI data from 2010 and 2011 by requesting data records from seven states: California (CA), Iowa (IA), Minnesota (MN), North Carolina (NC), New York (NY), Texas (TX), and Wisconsin (WI). Additional data from Nebraska (NE) was compiled from 2011. These eight states were selected for sampling using several criteria in consultation with industry experts. First we selected states that represented the largest concentrations of domestic swine production (i.e. Iowa, North Carolina, and Minnesota) based on National Agricultural Statistics Service (NASS) data. This ensured that our sampling would capture the core network of domestic swine shipments. To ensure that we also characterized the periphery of the network, we selected states that represented different production types or were geographically distant from the integrated domestic swine production systems in Iowa and North Carolina. These eight states represent 36% of all swine operations, 63% of all animals in production, and account for approximately $12 billion in production value^1^.

ICVI data are maintained for regulatory purposes by both the state where the shipment originated and the destination state. We, therefore, requested that states send ICVI data for shipments leaving the state to avoid duplicate records. To construct a digital database, we entered information from a 30% systematic sample of paper records from each year based on minimum sampling suggestions^29^. We entered the following information included in the ICVIs: the origin and destination address of the shipment, the dates the animals were shipped; the purpose of the shipment; the number of head; and the breed, age, and sex distribution of the swine in the shipment. The address data were converted to latitude and longitude coordinates using standard geocoding methodology. We cleaned the data by removing shipments with missing address information and shipments reporting no swine. The resulting database contained 6,751 shipments of over 3.3 million head. There were 4,346 unique premises locations from 1,004 unique origin or destination counties.

### Characteristics of swine shipments

To determine how well ICVI shipments represent the swine industry, we compared the age, sex, and production diversity captured in ICVI shipments to known characteristics of the industry. Specifically, we expect ICVIs to capture three features of the industry. First, we expect shipments for breeding and feeding to be larger and more common than shipments for show and sale. Second, we expect most breeding and feeding animals to originate from Iowa, Minnesota, Wisconsin and North Carolina because these states historically represent a large proportion of the swine industry. Third, we expect shipments to contain primarily young animals as weaned animals are shipped for feeding or finishing.

We compared the size of shipments (number of swine) by purpose with a Mann-Whitney (or Wilcoxon rank sum test). The Mann-Whitney test is a non-parametric test for differences in the median shipment size. We compared shipment size for breeding, feeding, sale, and show shipments and used a Bonferroni correction to account for multiple comparisons. For the Bonferroni correction, we adjusted the individual confidence level upward from at least 95% confidence to at least 100(1–0.05/k)% confidence, where k is set equal to 12 for the 12 tests comparing four production types each year. Thus, significant differences in shipment size occur when p-values are less than, p = 0.004. We tested if the proportion of breeding, feeding, sale, and show shipments is consistent between states in each year with Chi-square tests. We accounted for multiple comparisons with a Bonferroni correction, where p-values less than p-value = 0.0009 were considered significant (k = 53, for 46 tests comparing among seven states in 2010 and 2011, and seven tests comparing differences between years in each state). We also tested for differences between years in the proportion of swine in each age category. However, we do not test for differences between years in the proportion of swine that were male and female with Chi-square tests due to differences in reporting between years.

### Network construction and analysis of network characteristics

Network models summarize shipment patterns by representing the units of interest as nodes and shipments between them as edges. We aggregated the ICVI data at the county scale following previous analyses in cattle, such that each county represents a node in the network^18,19^. We also compared data aggregated at the state-level to determine if coarser scales remain descriptive. We constructed networks with nodes representing premises within a county to prevent identification of individual premises. Edges in the network represent the presence of directional shipments between two counties and can be weighted by the number of shipments or the number of swine moving between the counties. We constructed separate networks for swine shipments in 2010 and 2011 by aggregating the data across each year. For networks created with the 2011 data, we constructed one network based on data from all 8 states and another network without data from Nebraska for comparison with the data from 2010.

For each network, we calculated five node-level network measures and nine measures of network structure^12^ (Table 1). We also evaluate how much the sampling process influenced network characteristics. Specifically, ICVI data only record outgoing shipments that cross state lines and therefore, our dataset does not capture shipments originating from states not included in the dataset. To evaluate the consequences of this for network structure, we also constructed networks with nodes only in the states where out-going data were available. We provide a detailed consideration of the caveats, data availability, and sampling requirements to describe swine shipments in the discussion. We calculated network measures using the igraph package^30^ for R statistical software version 3.1.2^31^.

**Table 1:** Network analysis terms and their definition applied to swine shipments.

Name	Definition
**Node-level measures**	
in-degree	The number of unique counties that sent at least one shipment to the county in question.
out-degree	The number of unique counties that received at least one shipment from the county in question
weighted in-degree	The number of shipments or swine received by the county in question.
weighted out-degree	The number of shipments or swine sent from the county in question.
betweenness	The number of shortest paths between any two counties that go through the county in question.
**Network-level measures**	
number of nodes	The number of observed counties in the network. We use the number of nodes as a measurement of network size.
number of edges	The number of unique origin and destination pairs that were involved in at least one swine shipment. We calculate the number of edges on undirected networks.
density or connectance	The proportion of potential edges in the network that is actually present.
diameter	The maximum number of steps in the set of shortest paths between all county pairs.
giant strongly connected component (GSCC)	The largest set of counties in which a directed path exists between any counties in the set.
giant weakly connected component (GWCC)	The largest set of counties that are accessible to each other regardless of the direction of the edges between them.
assortativity	The correlation between a county’s total degree and the degree of the nodes connected to it. Assortativity ranges from −1 to 1, with positive values indicating that nodes with high or low numbers of shipments interact mostly with nodes with similar degree values.
transitivity	The ratio of the number of triangles and the number of connected triples in the network. Transitivity ranges from 0 to 1, with higher values indicating more connected subnetworks.
reciprocity	The proportion of edges where there is an edge in the opposite direction. Reciprocity ranges from 0 to 1 and is calculated as the proportion of county pairs connected by a shipment, where shipments travel in both directions.

### Evaluation of ICVI data: Comparison with National Agricultural Statistics Service data

The NASS Census collects information about the swine industry at the county scale every five years, providing a detailed inventory of breeding and market hogs. We use information on the number of premises per county in the 2012 NASS farm census as a standard to compare the ICVI dataset. Specifically, we define premises in the NASS dataset as all swine operations with the capacity to raise breeding or market swine.

To evaluate the ICVI data, we first examined how well it represents the underlying infrastructure of the industry in the eight states where both datasets were available (CA, IA, MN, NC, NE, NY, TX, and WI). The methods for this analysis follow those described in Buhnerkempe *et al*.^18^. Briefly, we calculated a proxy for the number of premises per county represented in the ICVI data as the number of unique locations sending or receiving a shipment. This proxy may undercount the number of premises due to missing or unusable address information on the ICVI forms or over-count the number of premises due to some premises reporting multiple addresses. We also expected the number of premises reported in the NASS dataset to be larger than the number of premises captured in the ICVI dataset because the ICVI dataset is a 30% sample and not all premises in the NASS dataset send or receive shipments across state lines. We, therefore, did not compare the number of premises identified in the two datasets, but considered both as proxies of the true number of farms and evaluated the correlation between them.

We tested for correlations between the number of premises in a county identified in the ICVI data and the 2012 NASS census with a generalized linear model, with quasi-Poisson errors and a log link function. We tested if the counties located on state borders are differently sampled compared to other counties. This could occur if border counties were more likely to ship across state lines. Thus, we consider the following independent variables: an indicator variable for whether a county is bordering another state, a factor for the effect of each state, a factor for the effect of each year, the number of farms per county in the NASS data, and all two-way interactions between them. We conducted model selection by backwards elimination based on quasi-Akaike Information Criterion (qAIC). qAIC is a measure of model fit and parsimony similar to Akaike Information Criterion (AIC) and is appropriate for quasi models^32,33^. We started with a full model including all potential independent variables and sequentially removed the interaction terms and then the main effect terms if qAIC was reduced. The final model was selected when no additional terms could be dropped. We argue that the ICVI dataset captures the underlying infrastructure of the industry if there is a positive association between the NASS and ICVI datasets that is consistent in both years. Further information on model selection, the statistical model, and parameter inference following model selection are provided in Supplement A.

We also tested for correlations between county-level network properties (in-degree, weighted in-degree, out-degree, weighted out-degree, and betweenness) and measures of industry infrastructure provided in the NASS census. Specifically, we compared three measures of county-level premises frequency and three measures of animal frequency from the 2012 NASS farm census to each network metric. County-level premises frequency data represent the number of farm operations with domestic swine inventory, the number of farm operations with breeding swine, and the number of farm operations with production swine. The county-level animal frequency measures included the total number of swine in inventory, the number of breeding swine, and the number of production swine. NASS data are only available for counties with greater than five farms to protect farm privacy; this analysis only used counties where NASS data are available.

## Results

### Characteristics of swine shipments

**Figure 1:**
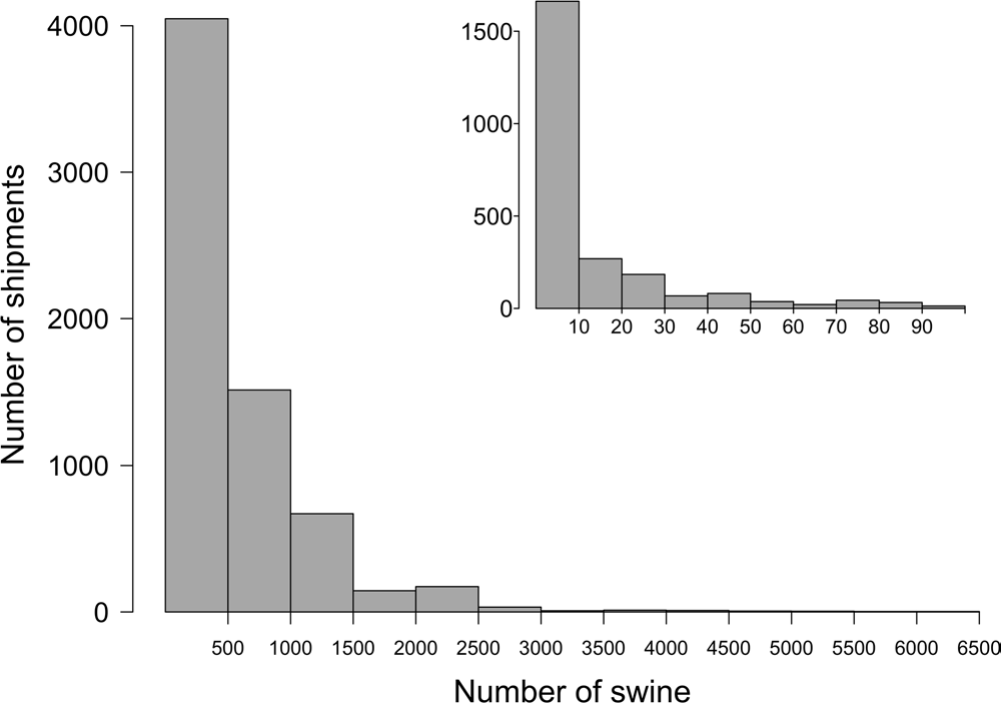
Size histogram of shipments captured by ICVIs in 2010 and 2011 from California, Iowa, Minnesota, New York, North Carolina, Texas, and Wisconsin. The inset figure shows the distribution of shipments containing less than 100 swine.

Swine shipments represented in the ICVI data primarily contained a small number of animals, but the distribution of swine per shipment was highly skewed (Fig. 1). The median shipment size was 330 head, the mean shipment size was 503 head, and shipment size ranged from 1 to 6,500 head. The median number of swine per shipment was higher in 2010 compared to 2011, with a median of 380 head in 2010 and 260 head in 2011 (Mann-Whitney test, p = 0.0003).

**Figure 2:**
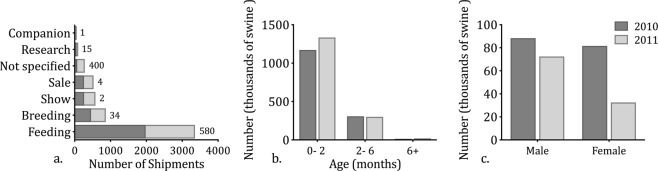
Demographic and production characteristics of ICVI swine shipments. (**a**) Barplot of the number of shipments by purpose for 2010 and 2011. Numbers at the end of each bar represent the median shipment size for each purpose across both years. (**b**) The age and (**c**) sex of swine shipped in 2010 and 2011. Data for each year includes the following states: California, Iowa, Minnesota, New York, North Carolina, Texas, and Wisconsin. Figure B1 displays the production characteristics for each state; Figure B2 displays demographic information including data from Nebraska for 2011.

Shipments for feeding purposes were the most common and largest type of shipment (Fig. 2a; Supplement B, Fig. B1). The median size of feeding shipments was 580 head. Shipments for breeding, sale, and show were also common, but they were smaller than feeding shipments (Fig. 2a). These trends were consistent between years (Supplement B, Table B1). When states were compared separately, shipments from Iowa, Minnesota, North Carolina, and Wisconsin were predominantly for feeding purposes while shipments from Texas, New York, and California were predominantly for sale purposes (Table 2). Accordingly, we identified significant differences in the proportion of shipments by purpose among states. These state-level heterogeneities were consistent between 2010 and 2011 (Supplement B, Table B2, Fig. B1).

**Table 2:** Summary of the number of shipments, number of swine, and production types for swine shipments in each state and year.

Origin State	2010								
	CA	IA	MN	NC	NEEE	NY	TX	WI	Total
No. Shipments	81	949	1,046	542	—	80	101	311	3,110
No. Head	1,517	433,641	573,578	511,106	—	679	1,542	86,546	1,608,609
Head/Shipment	2	360	498	800	—	6	6	100	380
Max Head/Shipment	440	5,200	5,000	6,500	—	40	90	3,200	6,500
% Breeding	1.2	11.0	17.9	17.9	—	0.0	1.0	20.9	14.6
% Feeding	0.0	70.4	70.2	78.4	—	0.0	1.0	49.2	63.7
% Sale	55.6	1.7	4.0	0.7	—	83.8	48.5	12.2	8.4
% Show	21.0	13.7	3.4	0.7	—	15.0	26.7	11.9	8.5
	**2011**								
No. Shipments	77	910	766	314	832	86	257	280	3,522
No. Head	1,762	408,155	430,799	230,266	503,806	745	69,222	80,048	1,724,803
Head/Shipment	2	328	380	625	550	6	3	150	260
Max Head/Shipment	220	6,500	6,420	5,000	4,200	40	5,300	2,500	6,500
% Breeding	0.0	13.8	21.8	22.9	27.4	1.2	1.9	15.0	18.2
% Feeding	0.0	58.7	61.0	71.0	57.0	0.0	9.7	49.3	52.8
% Sale	61.0	0.1	2.3	1.9	0.4	81.4	26.8	16.8	7.6
% Show	22.1	15.8	4.0	1.6	7.9	11.6	24.5	16.9	10.9

The median number of head per shipment is represented by Head/Shipment while the maximum number of head per shipment is represented by Max Head/Shipment.

Most shipments consisted of swine less than 2 months of age (Fig. 2b; Supplement B, Fig. B2). There were 425 (7%) ICVI records that did not report information on sex. However, based on the 93% of shipments reporting sex, a higher number of female swine were moved (Fig. 2c). Differences in the number of male and female swine shipped in 2010 compared to 2011 may be driven by reporting biases, as 6% of shipments were missing information on sex in 2010 and 9% of shipments were missing information on sex in 2011.

### Network characteristics

The swine network consisted of 676 counties from 45 states that either sent or received shipments in 2010 and 725 counties from 48 states in 2011 (Table 3). When Nebraska was also considered in 2011, the network contained 809 counties from 48 states. For both years, networks showed low levels of connectedness: 12% and 13% of counties belonged in the GSCC and 95.0% and 94% of counties belonged in the GWCC for 2010 and 2011, respectively (Supplement B, Fig. B3). Network reciprocity was low, indicating that counties rarely both send and receive shipments from each other. Networks from both years also had low transitivity, low assortativity. These properties indicate that counties connected to a common node are equally likely to themselves be connected, and counties with high degree values are equally likely to be connected to counties with high and low degree values. Few network characteristics were influenced by how the network was sampled (Supplement B, Table B3). When networks were constructed to only include nodes from the states with data, we observed higher density and higher assortativity compared to networks based on all nodes.

**Table 3:** Properties of networks constructed with nodes as counties or states.

	County Scale			State Scale		
	2010	2011	2011 (+NE)	2010	2011	2011 (+NE)
Number of nodes	676	725	809	45	48	48
Number edges	1364	1345	1709	136	154	175
Number of shipments	3110	2690	3522	3110	2690	3522
Diameter	9	11	12	4	3	4
GSCC size	80	70	107	7	7	8
GWCC size	643	680	761	45	48	48
Density	0.006	0.005	0.005	0.14	0.14	0.16
Assortativity	−0.11	−0.15	−0.11	−0.87	−0.87	−0.89
Transitivity	0.02	0.01	0.03	0.28	0.26	0.29
Reciprocity	0.05	0.05	0.04	0.37	0.44	0.38

Separate networks were constructed for shipments from 2010 and 2011. Because additional data were available for Nebraska in 2011, we created networks with (2011 +NE) and without (2011) shipments from this state, to explore the consequences of the additional data on network structure.

Networks constructed at the state-level were smaller and comparatively well connected compared to networks constructed at the county scale (Table 3). Network density was high and indicates that 14–16% of the possible edges between the states studied were realized, and that all states with data were in the GSCC. Network transitivity, and reciprocity were also high. This indicates high levels of clustering and bidirectional shipments between states. High, negative, assortativity values indicate that states with high numbers of shipping partners interact mostly with states with low numbers of shipping partners.

### Node-level characteristics

**Figure 3:**
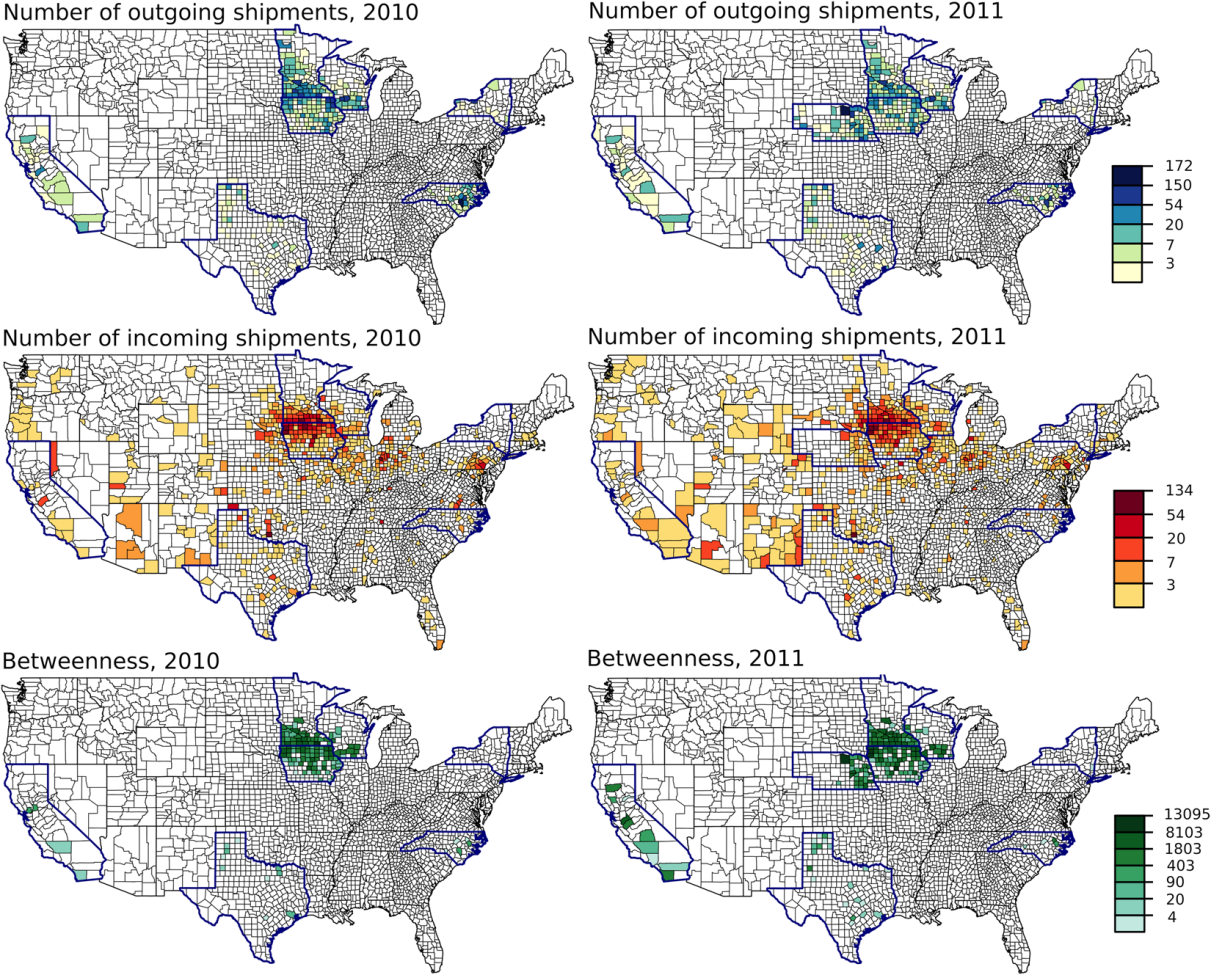
County-level patterns of weighted out-degree (number of outgoing shipments), weighted in-degree (number of incoming shipments), and betweenness. Maps in the left column are based on ICVI data from 2010 while maps in the right column are based on ICVI data from 2011. Colors represent the data on the log scale. Data were available from states outlined in dark blue.

**Figure 4:**
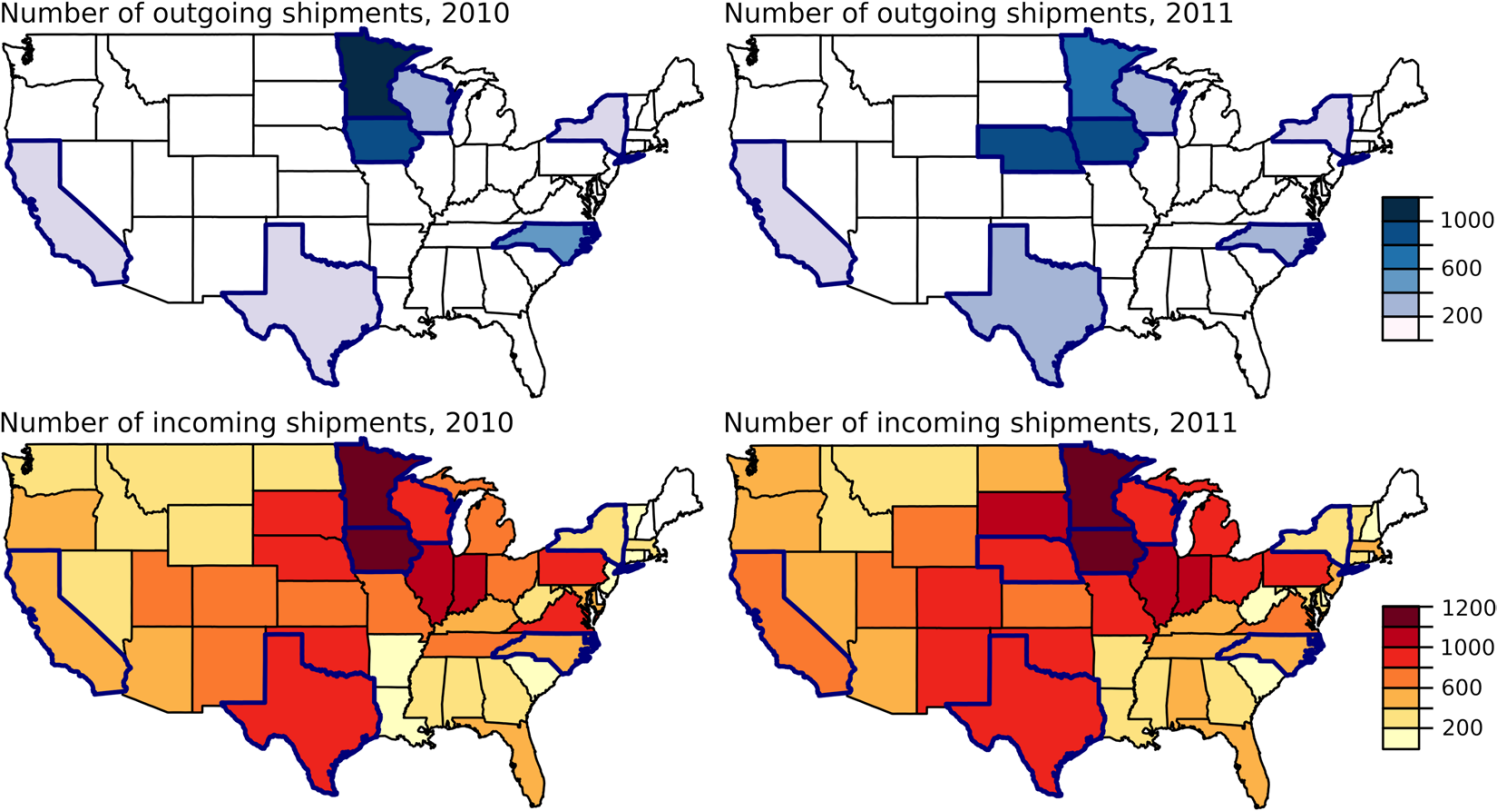
State-level patterns of weighted out-degree and weighted in-degree in 2010 and 2011. Colors indicate the volume of shipments either sent or received by a state. In the top row, states colored in white did not send a shipment while in the bottom row states colored in white did not receive a shipment. Data were available from states outlined in dark blue.

At the county scale, each county was connected to a median of 4 other counties in both 2010 and 2011 (mean of 8). A few counties were responsible for most of the connections. The maximum number of connections observed was 108 in 2010 and 98 in 2011. The maximum number of shipments sent from a county was 172 in 2010 and 167 in 2011. Most shipments were received in the central United States. Counties in Iowa and southern Minnesota, consistently had the highest levels of in-degree (Fig. 3a,b). Counties in Iowa, eastern Nebraska, and southern Minnesota also had the highest levels of out-degree (Fig. 3c,d) and betweenness (Fig. 3e,f). However, we did not consider data from other states, so we cannot compare the relative number of shipments leaving other central states. At the state scale, each state was connected to a median of 8 other states in 2010 and 9 other states in 2011 (mean of 12 and 14, respectively). The movement patterns identified at the county-scale were also observed at the state scale. Iowa and Minnesota consistently had high levels of in-degree while Iowa, Minnesota, and Nebraska had high levels of out-degree (Fig. 4).

### Evaluation of ICVI data: Comparison of farm counts in the ICVI and NASS data

The best model predicting the number of farms per county in the ICVI data included the following independent variables: the number of farms counted in the NASS data, state identity, whether the county was on a state border, and the interaction between state identity and the number of farms in the NASS data (Supplement A, Table A1). There was an overall positive association between the number of farms identified in the NASS and ICVI datasets (Supplement A, Fig. A1). This positive association was consistent in both 2010 and 2011. The number of farms identified in the ICVI data also varied by state, with Iowa consistently having the highest and Wisconsin having the lowest average number of farms per county. However, the strength of the association between the number of farms in the NASS and ICVI datasets varied by state. For example, in both years, significant positive associations occurred in Iowa, Minnesota, Nebraska, North Carolina and Wisconsin, but no association was found in California, Texas, and New York (Supplement A, Table A2, Fig. A1). Parameter values and significance tests are provided in Supplement A.

### Evaluation of ICVI data: Correlations between network properties and NASS data

**Figure 5:**
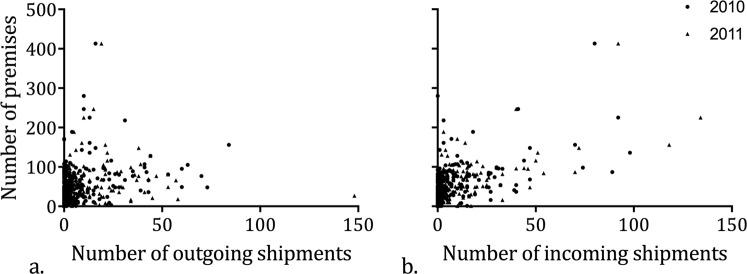
There was a positive association between the number of premises per county in the 2012 NASS data and both the number of outgoing shipments and the number of incoming shipments in 2010 and 2011.

Network properties, such as in-degree, the number of incoming shipments (weighted in-degree), out-degree, the number of outgoing shipments (weighted out-degree), and betweenness were positively associated with each measure of industry infrastructure provided in the NASS census (Supplement A, Table A3). The strongest correlations occurred between weighted or unweighted in-degree and the total number of premises, the total number of premises with production, the total number of swine per county, and the total number or swine per county for production. NASS measurements were more tightly correlated with in-degree than out-degree in both years (Fig. 5). However, stronger correlations occurred in 2011 compared to 2010. When broken down by production and breeding premises, production premises consistently predicted network in-degree better than breeding premises, while the number of breeding swine most strongly predicted out-degree (Table A3).

## Discussion

Shipment characteristics derived from the ICVI data are consistent with a vertically integrated domestic swine industry in which large numbers of animals are shipped to the mid-western states of Iowa, Nebraska, and Minnesota for feeding and slaughter^3^. The counties that we identified as receiving the highest volumes of domestic swine based on high in-degree values were also the counties with established feeding infrastructure based on the NASS data. However, we discuss a number of inconsistencies between our results and features of the domestic swine industry that highlight aspects of the industry that are not well understood but may be important for disease risk and allocation of surveillance resources.

The vertical integration of the U.S. domestic swine industry is apparent from network statistics that describe a well-connected, centrally located industry. The high proportion of counties observed in the GWCC (95–96%) and a small proportion of counties in the GSCC (12–13%) indicates a well-connected industry with a small number of counties that both send and receive shipments from one another. The county network’s low reciprocity and low, negative assortativity indicates that the network primarily consists of shipments moving in a single direction to large hubs and that groups of counties that send shipments back and forth are rare. Counties sending large numbers of shipments occurred heterogeneously in North Carolina, California, and the central states. In contrast, counties receiving large numbers of shipments and counties with high betweenness were primarily located in the central United States (northern Iowa, eastern Nebraska and southern Minnesota). This cluster of central counties was stable between years and is consistent with an industry where shipments are sent to the central United States for feeding. These features – well-connected networks with low, negative assortativity and spatial clusters of farms— are consistent with farm-level networks of swine shipments in France^34^, Germany^35^, and Sweden^14^.

These features of the U.S. swine industry may have important implications for disease transmission and surveillance given the importance of animal shipments in many disease outbreaks^4,7,36^. For example, states receiving high numbers of shipments are associated with a higher risk for porcine epidemic diarrhoea virus (PEDV^9^) and porcine reproductive and respiratory syndrome outbreaks (PRRSV^10^), and the spatial transmission of human-origin swine influenza is associated with swine shipments^24^. For certain parts of the U.S. domestic swine industry, measurements characterizing a node’s centrality to the network (e.g. degree, betweenness) may provide a first approximation for assigning potential importance in disease spread and in turn surveillance. Betweenness is particularly useful for capturing global network structure because it quantifies a node’s role in the flow of animals through the entire network^12^. Betweenness has been useful for predicting disease outcomes for U.K. cattle movement networks^37^ and identifying market hubs^38^. Disease spread simulations targeting counties with high centrality result in significantly reduced disease spread^39^. It has also been proposed as a useful metric for ranking a node’s importance in disease spread for cattle movement networks (United States^23^; United Kingdom^40^). In our analyses, when counties are ranked by betweenness, four states accounted for the top ten counties and most of the top 100 counties (Fig. 3).

Despite this hub-like nature of the swine shipment network, our results identified a poor association of out-degree with NASS metrics, indicating that parts of the industry captured by ICVI data may be more dispersed and less connected than previously thought^3^. Many counties receiving shipments were also not associated with the feeding infrastructure in the upper central states. This dispersed geographic distribution of county-level in-shipments across many regions in the western United States was qualitatively associated with feeding infrastructure (Fig. 5). This may indicate that ICVI shipments capture parts of the industry that are less connected to the GSCC in the central United States but still receive shipments from the core of the network.

While we believe that ICVI data have utility for supporting the development of surveillance strategies and identifying counties of potential importance for disease spread, they do have limitations for inference at the national-scale for management of disease. ICVIs are designed to capture interstate shipments and, similar to previous studies of cattle shipment networks in the United States^18^, these analyses’ strength lies in capturing longer-distance swine shipments. To date, these long distance, county-level shipments are missing from other network studies of domestic swine^9,10^. However, the ICVI network is limited in that we did not sample all states with domestic swine industries, we use a 30% sample of ICVI shipments, and the ICVI dataset does not include shipments to slaughter or shipments that are permitted under other regulatory mechanisms, discussed below. Despite these limitations, our sample is consistent with other descriptions of the domestic swine industry, indicating that it likely represents the largest components of the industry and the general agreement among years in our sample indicates that our characterization may provide inference across years. Frameworks are available for scaling up the 30% sample to represent all shipments^41^, but data thinning simulations suggest that network structure and disease processes can be estimated directly from the data with at least a 30% sample of edges^29^.

These data limitations underscore the need for swine shipment data to be more widely available. In addition to ICVI data, swine may also be shipped under agreements for interstate movement within production systems (sometimes referred to as commuter agreements). These agreements are developed for swine production systems (multiple sites of swine production that are connected by ownership or contractual relationships) that allow the movement of swine between premises within the production system without individual identification. Before this work, however, the volumes and types of shipments captured in either dataset were unknown. We provide summary information from the state-level networks based on ICVI data from 2010 and 2011 as supplementary material. Improved data availability would enable comparisons between ICVI-based shipments and shipments within a production system across years. This work will be particularly important for industries that have gone through significant changes in structure, such as the U.S. domestic swine industry.

## Conclusion

Characterizing national scale domestic swine movements has important implications for risk analysis to support disease preparedness and is one of the most important applications of our analyses. We have identified counties (i.e. nodes) that are highly connected based on having high degree and betweenness values that may be of importance for improving targeted surveillance. These highly connected nodes may also indicate locations where disease controls such as enhanced surveillance could improve disease response during an outbreak. Future work using disease simulation models in conjunction with this empirical network could investigate maintaining transportation channels for unaffected producers, optimizing disease surveillance, and evaluate outbreak mitigations providing significant benefits for understanding optimal control actions during an outbreak that both improve control and reduce impacts to the swine industry.

## Supplementary information

Supplementary Information

## Data Availability

The datasets analyzed during this study are available from the United States Department of Agriculture (USDA), but restrictions apply due to confidentiality concerns about farm demographics and locations. They were used under a Memorandum of Understanding for the current study and are not publicly available. Data, however, are available from USDA authors, Dr. Katie Portacci (Katie.Portacci@aphis.usda.gov) and Dr. Ryan Miller (Ryan.S.Miller@aphis.usda.gov) upon reasonable request, in compliance with Federal regulations, and under certain agreements with the United States Department of Agriculture.
