# Supplementary material for: Spatio-temporal patterns and characteristics of swine shipments in the U.S. based on Interstate Certificates of Veterinary Inspection
Source: Sci Rep. 2019 Mar 8;9:3915. doi: 10.1038/s41598-019-40556-z (PMC6408505; doi:10.1038/s41598-019-40556-z)
Supplement: Supplementary file 1 — Supplementary Information [file 41598_2019_40556_MOESM1_ESM.pdf]

Supplementary Material:

Spatio-temporal patterns and characteristics of swine shipments in the U.S. based on Interstate Certificates of Veterinary Inspection

Erin E. Gorsich[1,2,3,4¶*], Ryan S. Miller[1,5¶], Holly M. Mask[1], Clayton Hallman[1,5], Katie Portacci[5], Colleen T. Webb[1,2]

1. Department of Biology, Colorado State University, Fort Collins, CO, USA
2. Graduate Degree Program in Ecology, Colorado State University, Fort Collins, CO, USA
3. The Zeeman Institute: Systems Biology and Infectious Disease Epidemiology Research (SBIDER), University of Warwick, Coventry, UK
4. School of Life Sciences, University of Warwick, Coventry, UK.
5. USDA APHIS Veterinary Services, Center for Epidemiology and Animal Health, Fort Collins, CO, USA.

¶ Authors contributed equally to this work

*corresponding author: eringorsich@gmail.com

Supplement A - Comparison of ICVI data with NASS data

Table A1. Model selection table for comparison of the number of farms in the ICVI data (y_i^{cvi}) with the number of farms in the NASS data ($NASS_i$). The full model includes 31 parameters in the following statistical model:

$$\ln\left(y_i^{cvi}\right) = \beta_o + \beta_1 \text{NASS}_i + \beta_{2,j}\text{state}_j + \beta_3 \text{border}_i + \beta_4 \text{ year } + \beta_{(1,2),j}\text{NASS}_i * \text{state}_\text{j} + \beta_{(1,3)}\text{NASS}_\text{i} * \text{border}_\text{i} + \beta_{(1,4)}\text{NASS}_i * \text{ year } + \beta_{(2,3),j}\text{state}_j * \text{border}_i + \beta_{(2,4),j}\text{state}_j * \text{year } + \beta_{(3,4)}\text{border}_i * \text{year} + \varepsilon_i$$

In this model, the 31 parameters include 1 parameter representing the relationship between the number of farms in the ICVI and NASS data (β_1); 6 parameters representing the relationship between the average number of ICVI farms in each state ($\beta_{2,j}$); 1 parameter representing the difference between the median number of farms in counties bordering state lines compared to non-border counties (β_3); 1 parameter representing the difference between the median number of farms in 2011 compared to 2010 (β_4); and 21 parameters capturing the two-way interactions between each predictor variable ($\beta_{(1,2),j}, \beta_{(1,3)}, \beta_{(1,4)}, \beta_{(2,3),j}, \beta_{(2,4),j}, \beta_{(3,4)}$). In the model selection table below, we abbreviate these parameter combinations as nass, border, st, and yr for parameters $\beta_1, \beta_{2,j}, \beta_3$, and β_4. We conducted model selection and inference based on the data with one county excluded because the NASS data reported 413 farms in this county. The number of parameters (np), qAIC, and change in qAIC values associated with each model selection step (ΔqAIC) are also displayed.

Model	np	qAIC	ΔqAIC
**Number of Farms per county in the ICVI data ~**			
nass+ st + border+ yr+ nass*st + nass*border+ nass*yr + st*border + st*yr **+** border*yr	31	1557	16
nass+ st + border+ yr+ nass*st + nass*border+ nass*yr + st*border + border*yr	25	1551	10
nass+ st + border+ yr+ nass*st + nass*border+ nass*yr + border*yr	19	1547	6
nass+ st + border+ yr+ nass*st + nass*yr + st*border	18	1545	4
nass+ st + border+ yr+ nass*st + nass*yr	12	1543	2
nass+ st + border+ yr+ nass*st	11	1543	2
nass+ st + border+ nass*st	10	1541	-

Table A2. Parameter table. Parameter estimates, standard error (SE), and statistical tests (T-values and p-values) associated with the best model predicting the number of farms per county in the ICVI data. We note that statistical inference based on the best-fitting model following model selection may result in type I errors because the hypothesis tests assume a single model[1,2] and do not consider uncertainty in model selection[3]. We, therefore, interpret test results from the full model (full, see table A1). We display the best-fit model to show its overall fit (Figure A1) and consistencies in inference between the two models. The parameter estimate for each state compares the median number of farms per county in the that state compared to the median number of farms per county in North Carolina; the parameter estimate for the border indicator compares the median number of farms per county in boarder counties to non-border counties

Parameter	Estimate	SE	T-value	p-value	full
**Number of Farms per county in ICVI data**					
NASS farm count	0.009	0.001	7.616	<0.0001*	<0.0001*
Border Indicator	0.397	0.057	6.915	<0.0001*	0.713
California	0.170	0.319	0.533	0.594	0.942
Iowa	0.812	0.169	4.807	<0.0001*	0.019
Minnesota	0.613	0.182	3.364	0.001	0.268
New York	0.147	0.441	0.333	0.739	0.610
Texas	-0.030	0.217	-0.136	0.892	0.177
Wisconsin	0.141	0.267	0.528	0.598	0.920
California× NASS farm count	-0.002	0.007	-0.299	0.765	0.783
Iowa × NASS farm count	0.001	0.001	1.005	0.315	0.289
Minnesota × NASS farm count	0.008	0.002	4.696	<0.0001*	<0.0001*
New York × NASS farm count	-0.012	0.009	-1. 319	0.188	0.239
Texas × NASS farm count	-0.003	0.004	-0.817	0.414	0.473
Wisconsin × NASS farm count	0.005	0.004	1.272	0.204	0.189

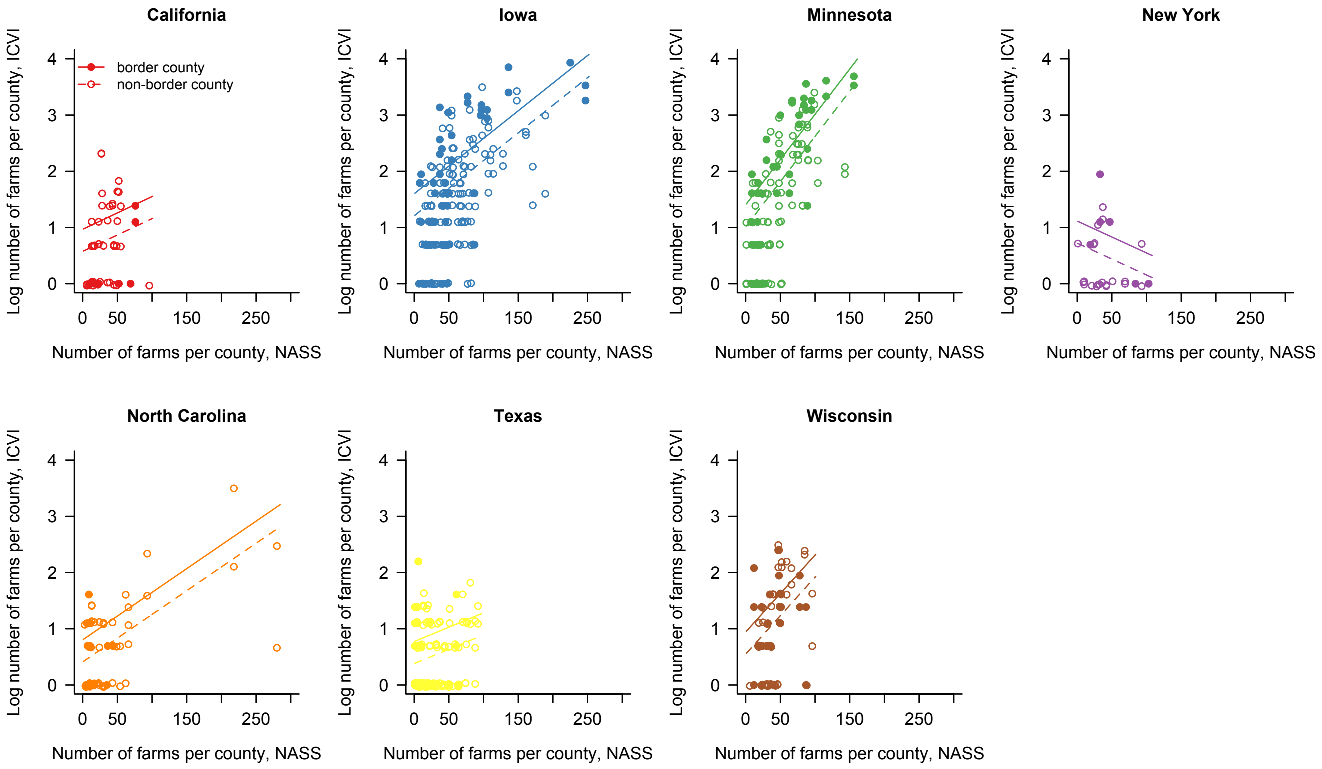

Fig. A1. Data and model-fits for the final model presented in Table B2. Solid lines and filled circles represent the predictions and data for counties that border state lines. Dashed lines and open circles represent the predictions and data for counties that do not border state lines.

Table A3. Correlations between network measures and industry information in NASS.

	Number of premises			Inventory of swine		
	Total	Breeding	Production	Total	Breeding	Production
**2010**						
In-degree	0.56 (0.48, 0.64)	0.20 (0.08, 0.31)	0.54 (0.42, 0.64)	0.45 (0.35, 0.55)	0.20 (0.07, 0.32)	0.18 (0.01, 0.34)
In-shipments	0.53 (0.43, 0.61)	0.18 (0.07, 0.30)	0.49 (0.36, 0.60)	0.47 (0.37, 0.56)	0.21 (0.08, 0.33)	0.20 (0.04, 0.36)
Out-degree	0.35 (0.24, 0.45)	0.20 (0.08, 0.31)	0.23 (0.08, 0.37)	0.34 (0.23, 0.44)	0.35 (0.22, 0.46)	0.13 (-0.04, 0.29)
Out-shipments	0.30 (0.19, 0.41)	0.16 (0.03, 0.27)	0.22 (0.07, 0.36)	0.33 (0.22, 0.44)	0.39 (0.27, 0.49)	0.14 (-0.03, 0.29)
Betweenness	0.38 (0.28, 0.48)	0.17 (0.05, 0.28)	0.33 (0.19, 0.46)	0.33 (0.21, 0.43)	0.26 (0.14, 0.38)	0.12 (-0.05, 0.29)
**2011**						
In-degree	0.66 (0.60, 0.79)	0.26 (0.15, 0.36)	0.73 (0.15, 0.36)	0.66 (0.59, 0.71)	0.44 (0.35, 0.54)	0.65 (0.54, 0.73)
In-shipments	0.63 (0.56, 0.69)	0.24 (0.13, 0.34)	0.67 (0.58, 0.74)	0.66 (0.59, 0.72)	0.45 (0.35, 0.54)	0.63 (0.53, 0.72)
Out-degree	0.27 (0.16, 0.37)	0.14 (0.03, 0.25)	0.19 (0.05, 0.32)	0.33 (0.23, 0.42)	0.45 (0.35, 0.54)	0.21 (0.05, 0.36)
Out-shipments	0.20 (0.10, 0.31)	0.09 (-0.02, 0.20)	0.14 (0.00, 0.28)	0.28 (0.18, 0.38)	0.44 (0.34, 0.54)	0.19 (0.03, 0.34)
Betweenness	0.51 (0.44, 0.60)	0.25 (0.15, 0.35)	0.48 (0.36, 0.58)	0.52 (0.44, 0.60)	0.42 (0.32, 0.52)	0.47 (0.33, 0.58)

Supplement B - Swine shipments

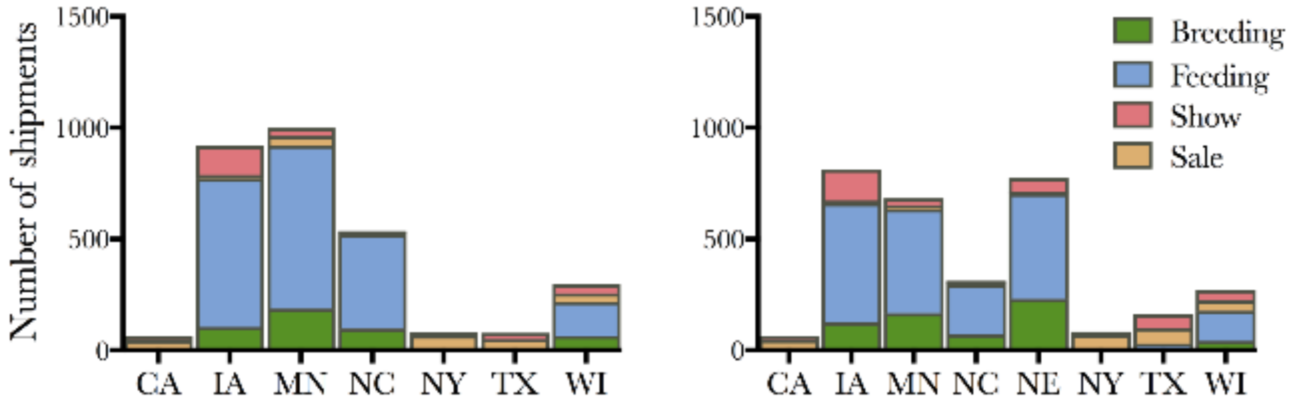

Fig. B1. Barplot of number of shipments by purpose for 2010 and 2011 for each state.

Table B1. Results of statistical tests to determine if shipment size varies by purpose. The data used for these analyses includes all 7 states in 2010 and 8 states in 2011. Values represent p-values from Wilcoxon signed rank tests. Significant differences occur at p-values less than p-value= 0.004.

2010	Breeding	Feeding	Sale	Show	Median Size
Breeding	-	<.0001	<.0001	<.0001	40
Feeding	-	-	<.0001	<.0001	600
Sale	-	-	-	<.0001	5
Show	-	-	-	-	2
2011					
Breeding	-	<.0001	<.0001	<.0001	27
Feeding	-	-	<.0001	<.0001	640
Sale	-	-	-	<.0001	4
Show	-	-	-	-	2

Table B2. Results of statistical tests to determine if shipment the proportion of shipments for breeding, feeding, sale, and show differ between states. Significant differences occur at p-values less than p-value= 0.0009.

				**2010**				
	CA	IA	MN	NC	NE	NY	TX	WI
CA	-	0.0005	0.0005	0.0005	-	-	**0.687**	0.0005
IA	-	-	0.0005	0.0005	-	0.0005	0.0005	0.0005
MN	-	-	-	0.0005	-	0.0005	0.0005	0.0005
NC	-	-	-	-	-	0.0005	0.0005	0.0005
NY	-	-	-	-	-	-	**0.006**	0.0005
TX	-	-	-	-	-	-	-	0.0005
				**2011**				
CA	-	0.0005	0.0005	0.0005	0.0005	**0.048**	0.0005	0.0005
IA	-	-	0.0005	0.0005	0.0005	0.0005	0.0005	0.0005
MN	-	-	-	**0.115**	0.0005	0.0005	0.0005	0.0005
NC	-	-	-	-	0.0005	0.0005	0.0005	0.0005
NE	-	-	-	-	-	0.0001	0.0005	0.0005
NY	-	-	-	-	-	-	0.0005	0.0005
TX	-	-	-	-	-	-	-	0.0005
2010 vs 2011	**0.917**	**0.005**	**0.013**	**0.053**		**0.664**	**0.002**	**0.067**

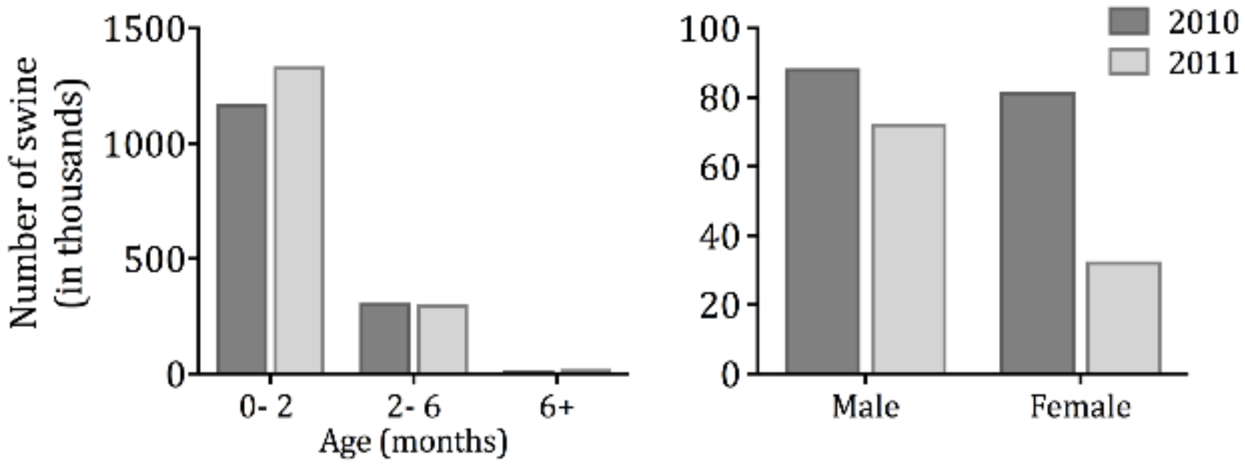

Fig. B2. Age and sex patterns remain consistent when data from Nebraska is included for 2011 compared to figure 3.

Table B3. Properties of networks constructed with nodes only in the states where out-going data were available (California, Iowa, Minnesota, North Carolina, Nebraska (NE), New York, Texas, and Wisconsin). Separate networks were constructed for shipments from 2010 and 2011 and for nodes at the state and county scale. Because additional data were available for Nebraska in 2011, we created networks with (2011 +NE) and without (2011) shipments from this state, to explore the consequences of the additional data on network structure.

	County Scale			State Scale		
	2010	2011	2011 (+NE)	2010	2011	2011 (+NE)
Number of nodes	261	248	321	7	7	8
Number edges	695	600	901	17	17	22
Number of shipments	1830	1421	2123	1830	1421	2123
Diameter	8	11	12	3	3	3
GSCC size	80	70	107	7	7	8
GWCC size	233	238	309	7	7	8
Density	0.02	0.02	0.02	0.81	0.81	0.79
Assortativity	0.01	0.07	0.06	-0.96	-0.96	-0.90
Transitivity	0.02	0.02	0.05	0.79	0.83	0.81
Reciprocity	0.08	0.09	0.07	0.64	0.83	0.63

2010 **2011**

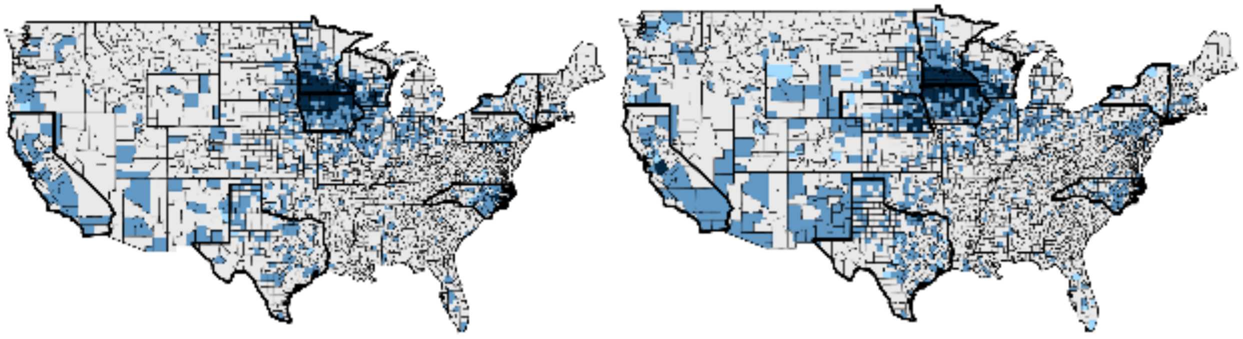

Fig. B3. Spatial patterns of the giant strongly and weakly connected components in 2010 and 2011 show a well-connected network. Counties in dark blue belong to the GSCC; counties in blue belong to the GWCC; counties in light blue were not part of either. Counties in light gray neither sent nor received a shipment in our dataset. Outgoing shipment data was collected from the states outlined in black.

References

1. Mundry, R. & Nunn, C. L. Stepwise Model Fitting and Statistical Inference: Turning Noise into Signal Pollution. *Am. Nat.* **173**, 119–123 (2009).
2. Freedman, D. A., Navidi, W. & Peters, S. C. On the Impact of Variable Selection in Fitting Regression Equations. *On Model Uncertainty and its Statistical Implications* (ed. Dijkstra, T. K.) 1–16 (Springer Berlin Heidelberg, 1988).
3. Burnham, K. P., Anderson, D. R. & Huyvaert, K. P. AIC model selection and multimodel inference in behavioral ecology: some background, observations, and comparisons. *Behav. Ecol. Sociobiol.* **65**, 23–35 (2011).

Supplemental Data Files

Edge-lists defining the network of swine shipments with nodes representing states in the U.S. and edges representing swine shipments in the ICVI data from either 2010 or 2011. Rows in the datasheets represent directed edges in the network. Columns in this file include: (1) origin.state = a two-letter abbreviation for the origin state of a directed edge; (2) destination.state = a two-letter abbreviation for the destination state of a directed edge; (3) origin.fips = a two-number abbreviation for the origin state of a directed edge (FIPS code); (4) destination.fips = a two-number abbreviation for the destination state of a directed edge (FIPS code); (5) number.shipments = the number of shipments sent between the origin and destination state (6) number.swine = the number of swine sent between the origin and destination state.

2010

origin.state	destination.state	origin.fips	destination.fips	number.shipments	number.swine
MN	NE	27	31	39	8512
NC	VA	37	51	56	21574
WI	OK	55	40	12	148
IA	MN	19	27	508	346814
MN	IA	27	19	805	518038
NC	IN	37	18	160	187120
NC	MS	37	28	3	77
NC	TX	37	48	17	398
WI	IA	55	19	161	69727
IA	IL	19	17	107	50298
WI	SD	55	46	6	52
NC	UT	37	49	18	429
NC	MO	37	29	8	261
MN	CO	27	8	13	4163
MN	WI	27	55	23	3299
MN	SD	27	46	77	28654
WI	OH	55	39	16	3337
WI	KS	55	20	14	1784
MN	MI	27	26	23	941
MN	IL	27	17	13	2533
WI	MN	55	27	38	391
WI	IN	55	18	14	39
WI	MI	55	26	1	2
NC	CO	37	8	2	51
WI	NE	55	31	3	2474
WI	PA	55	42	4	29
CA	WA	6	53	2	2
MN	NC	27	37	1	8
WI	ND	55	38	1	12
WI	NC	55	37	2	47
IA	MI	19	26	13	1973
MN	IN	27	18	4	19
MN	OK	27	40	13	876
NC	AZ	37	4	1	25
MN	PA	27	42	2	1795
MN	KS	27	20	8	1196
IA	WI	19	55	38	6238
IA	IN	19	18	32	7134
IA	WA	19	53	3	46
IA	KS	19	20	9	36
IA	TX	19	48	49	307
IA	SD	19	46	19	5130
IA	UT	19	49	3	17
IA	NM	19	35	3	6
MN	MO	27	29	6	343

IA	NE	19	31	35	9027
IA	ID	19	16	3	18
IA	OK	19	40	46	275
IA	CO	19	8	8	48
IA	MS	19	28	1	1
IA	OH	19	39	7	28
IA	OR	19	41	2	4
IA	MO	19	29	29	5023
IA	PA	19	42	3	217
MN	ND	27	38	3	2053
MN	TX	27	48	6	84
IA	GA	19	13	3	21
TX	OK	48	40	64	1405
IA	CA	19	6	15	775
IA	WY	19	56	4	42
MN	MD	27	24	1	2
TX	OH	48	39	2	2
TX	NY	48	36	3	3
TX	PA	48	42	2	2
TX	MI	48	26	1	1
TX	MA	48	25	2	2
TX	MD	48	24	1	1
TX	TN	48	47	1	1
NC	MN	37	27	14	15600
NC	IA	37	19	115	166783
NC	PA	37	42	86	66775
WI	IL	55	17	25	8406
NC	TN	37	47	27	24750
NC	AL	37	1	7	3712
WI	MD	55	24	1	8
NC	NE	37	31	10	7655
NC	SD	37	46	3	3300
NC	IL	37	17	5	4195
NC	GA	37	13	1	800
WI	FL	55	12	2	58
NC	OH	37	39	4	7400
MN	FL	27	12	1	680
NC	FL	37	12	4	169
IA	ND	19	38	1	4
IA	FL	19	12	2	64
MN	WA	27	53	1	22
CA	AZ	6	4	14	86
CA	CO	6	8	2	2
NY	OH	36	39	17	196
CA	IA	6	19	9	10
CA	NV	6	32	7	29

WI	TX	55	48	3	12
TX	NM	48	35	19	116
TX	NC	48	37	1	1
WI	NY	55	36	2	10
CA	OK	6	40	8	46
CA	MI	6	26	3	210
MN	OH	27	39	1	12
MN	MA	27	25	1	4
NY	PA	36	42	16	101
CA	UT	6	49	4	13
NY	MI	36	26	13	199
NY	MD	36	24	6	46
CA	MD	6	24	1	1
NY	NC	36	37	4	7
CA	OH	6	39	3	5
CA	OR	6	41	7	26
NY	IN	36	18	3	26
CA	HI	6	15	5	1003
NY	MA	36	25	3	11
CA	TX	6	48	11	65
NY	CA	36	6	2	7
NY	AZ	36	4	1	20
CA	KY	6	21	1	12
NY	FL	36	12	1	6
WI	KY	55	21	3	3
NY	CT	36	9	1	3
WI	CA	55	6	1	3
NY	WV	36	54	1	5
NY	TX	36	48	1	8
NY	OR	36	41	1	2
NY	UT	36	49	1	1
NY	NJ	36	34	2	9
NY	WA	36	53	1	20
CA	VT	6	50	1	1
CA	MO	6	29	2	5
WI	LA	55	22	1	1
NY	SC	36	45	1	3
IA	AR	19	5	2	24
IA	VA	19	51	1	2
MN	TN	27	47	1	1
MN	KY	27	21	1	1
CA	WV	6	54	1	1
MN	MT	27	30	3	342
NY	KY	36	21	4	7
NY	IL	36	17	1	2
NC	MD	37	24	1	32

TX	MO	48	29	3	6
WI	MO	55	29	1	3
IA	MT	19	30	1	9
IA	WV	19	54	1	5
TX	MN	48	27	1	1
TX	MS	48	28	1	1
IA	AZ	19	4	1	55

2011

origin.state	destination.state	origin.fips	destination.fips	number.shipments	number.swine
MN	SD	27	46	74	30650
WI	OH	55	39	19	3426
MN	MO	27	29	28	6698
MN	IA	27	19	530	354633
NC	VA	37	51	22	7428
MN	NE	27	31	34	13321
WI	MN	55	27	34	2007
IA	MN	19	27	476	304284
MN	CO	27	8	9	2577
NC	MO	37	29	34	538
NE	IA	31	19	360	267568
WI	IA	55	19	143	57143
IA	WI	19	55	46	7546
WI	NC	55	37	3	85
WI	SD	55	46	1	7
MN	NC	27	37	1	18
WI	KS	55	20	11	1780
WI	IN	55	18	11	1032
NE	MO	31	29	16	314
NE	MN	31	27	232	186069
WI	OK	55	40	11	230
NE	SD	31	46	61	37249
WI	IL	55	17	26	12359
WI	MI	55	26	3	1779
MN	KS	27	20	2	680
NE	KS	31	20	28	7705
MN	ND	27	38	11	4215
NC	UT	37	49	14	276
NC	TX	37	48	13	298
NE	OK	31	40	17	102
NC	CO	37	8	1	38
MN	WI	27	55	21	4135
NE	PA	31	42	3	57
NE	IL	31	17	12	279
MN	IL	27	17	10	3240
IA	IL	19	17	96	62430
IA	MI	19	26	10	3750
NY	OH	36	39	14	161
NC	AZ	37	4	1	57
NE	AZ	31	4	3	23
NE	WI	31	55	4	88
IA	NJ	19	34	1	10
IA	CA	19	6	17	472
IA	NE	19	31	43	14325
MN	MI	27	26	22	7103

IA	TX	19	48	32	209
IA	LA	19	22	5	12
NE	TX	31	48	22	130
IA	MD	19	24	2	3
IA	MT	19	30	1	1
NE	WY	31	56	14	92
MN	IN	27	18	7	702
TX	OR	48	41	4	5
IA	IN	19	18	33	4320
IA	OH	19	39	9	40
IA	PA	19	42	7	11
IA	UT	19	49	2	19
NE	CO	31	8	33	3846
NE	IN	31	18	6	20
NE	OH	31	39	1	2
TX	LA	48	22	2	2
IA	SD	19	46	14	5586
IA	MO	19	29	22	4352
WI	CO	55	8	1	2
IA	KS	19	20	10	137
IA	OK	19	40	25	162
IA	OR	19	41	5	55
IA	FL	19	12	2	2
NE	WA	31	53	1	5
NE	CA	31	6	2	6
IA	ID	19	16	4	6
NE	AR	31	5	1	28
TX	OK	48	40	70	16419
TX	IL	48	17	3	9
NE	MI	31	26	1	44
NE	OR	31	41	2	2
TX	UT	48	49	2	2
IA	CO	19	8	16	53
IA	WY	19	56	9	49
IA	NV	19	32	1	1
TX	NV	48	32	4	4
TX	SC	48	45	2	3
TX	TN	48	47	5	5
TX	MN	48	27	9	5032
TX	NJ	48	34	5	5
TX	PA	48	42	7	7
TX	NY	48	36	5	5
TX	MD	48	24	3	3
TX	WA	48	53	5	5
TX	VA	48	51	4	4
TX	NC	48	37	2	2

TX	NM	48	35	64	307
TX	GA	48	13	1	1
TX	AK	48	2	1	1
TX	CA	48	6	5	5
TX	MS	48	28	2	2
TX	WI	48	55	2	4
TX	MA	48	25	3	3
TX	AL	48	1	2	2
TX	FL	48	12	2	121
TX	SD	48	46	9	21095
NC	IN	37	18	104	125650
NC	PA	37	42	67	45666
NC	IA	37	19	17	20108
NC	MN	37	27	2	2350
NC	AL	37	1	7	3550
MN	OK	27	40	4	677
MN	TX	27	48	10	1737
NC	TN	37	47	5	2675
NC	MI	37	26	2	1400
NC	NJ	37	34	2	674
NC	MS	37	28	2	1350
NC	IL	37	17	1	500
TX	IA	48	19	17	26144
NC	OH	37	39	8	17600
IA	AR	19	5	1	1
IA	ND	19	38	1	1
IA	DE	19	10	1	1
IA	NM	19	35	7	38
IA	KY	19	21	7	76
MN	CA	27	6	2	412
NE	GA	31	13	1	27
NE	FL	31	12	3	128
IA	TN	19	47	1	10
IA	AZ	19	4	1	2
TX	OH	48	39	2	2
TX	MI	48	26	2	2
CA	IA	6	19	8	10
NY	GA	36	13	1	3
CA	MI	6	26	2	105
CA	IL	6	17	2	2
CA	AZ	6	4	9	53
CA	NV	6	32	6	21
NC	FL	37	12	5	95
IA	HI	19	15	1	185
NY	NJ	36	34	9	88
NY	WA	36	53	3	31

CA	KY	6	21	1	1
TX	AZ	48	4	1	2
CA	UT	6	49	4	28
CA	HI	6	15	8	1160
NY	PA	36	42	20	167
WI	WA	55	53	1	6
NY	MD	36	24	2	26
NY	OR	36	41	1	2
CA	IN	6	18	7	63
WI	TX	55	48	6	13
NY	MI	36	26	17	197
NY	NC	36	37	6	7
WI	NE	55	31	3	156
NY	AZ	36	4	1	13
CA	PA	6	42	1	1
NY	MA	36	25	6	30
NY	NH	36	33	1	1
NY	KS	36	20	1	3
NY	VT	36	50	1	2
CA	OH	6	39	3	3
CA	GA	6	13	2	3
CA	TX	6	48	10	90
CA	NC	6	37	1	1
WI	VT	55	50	1	2
CA	VA	6	51	1	1
CA	OK	6	40	8	198
CA	MN	6	27	1	1
WI	MS	55	28	1	7
NC	WV	37	54	1	1
TX	MT	48	30	2	2
TX	NE	48	31	2	2
WI	NY	55	36	1	4
WI	PA	55	42	1	1
IA	VT	19	50	1	1
NE	KY	31	21	6	14
NC	OK	37	40	2	3
MN	OH	27	39	1	1
NC	NY	37	36	1	1
NC	GA	37	13	1	1
IA	GA	19	13	1	5
NY	KY	36	21	1	1
NY	IN	36	18	1	8
NY	CT	36	9	1	5
CA	WA	6	53	1	4
CA	OR	6	41	1	2
WI	FL	55	12	1	5

TX	MO	48	29	4	10
NE	MT	31	30	1	3
NE	ID	31	16	2	5
WI	KY	55	21	2	4
TX	AR	48	5	2	3
TX	CT	48	9	2	2
NC	KY	37	21	2	7
CA	NM	6	35	1	15
